# The circRNA expression profile of colorectal inflammatory cancer transformation revealed potential predictive biomarkers

**DOI:** 10.18632/aging.204406

**Published:** 2022-11-29

**Authors:** Lu Lu, Yujing Liu, Guangtao Zhang, Yangxian Xu, Dan Hu, Guang Ji, Hanchen Xu

**Affiliations:** 1 Institute of Digestive Diseases, Longhua Hospital, Shanghai University of Traditional Chinese Medicine, Shanghai 200032, China; 2 Department of General Surgery, Longhua Hospital, Shanghai University of Traditional Chinese Medicine, Shanghai 200032, China; 3 Department of Internal Medicine, Shanghai Pudong New Area Hospital of Traditional Chinese Medicine, Shanghai 200032, China

**Keywords:** colorectal cancer, colorectal adenoma, circular RNAs (circRNAs), inflammatory cancer transformation, RNA sequencing, biomarkers

## Abstract

Colorectal cancer (CRC) is one of the most common malignant tumors in the world, and most colorectal cancer is transformed from colorectal adenoma (CRA). Identifying biomarkers for the early prediction of colorectal cancer would be an important finding. Circular RNA (circRNA) plays a key role in the occurrence and development of tumors, and its biological characteristics make it a potential biomarker for the early diagnosis of diseases. Therefore, we explored the relationship between circRNA and the malignant transformation from colorectal adenoma to colorectal cancer. We constructed inflammation-based tumorigenesis mouse models and performed high-throughput RNA sequencing to determine the expression profile of circRNAs in tissues at different stages of disease. Subsequent STEM analysis showed that with the development of the disease, 30 circRNAs were significantly downregulated, and 10 circRNAs were significantly upregulated. After qRT-PCR and Fish analysis verification, it was clear that mmu_circ_0008035 and mmu_circ_0000420 were significantly and continuously overexpressed in the development of colorectal cancer in our mouse model. Next, through homology analysis of circRNA in human and mouse and validation of clinical normal tissues, adenoma tissues and CRC tissues, we found biomarkers of has_circ0101338 ahashsa_circ0022426 that could predict the malignant transformation of human colorectal inflammation into CRC and have certain diagnostic value. In conclusion, our results may shed light on the mechanism of progression from precancerous adenoma to cancer and provide biomarkers that may be used in the early diagnosis of CRC.

## INTRODUCTION

Colorectal cancer is one of the most common malignant tumors and seriously influences people’s health and quality of life. It is the main cause of cancer death, with the third highest mortality rate of all malignant tumors according to recent research [1]. However, statistical analysis from 2008 through 2017 showed that CRC death rates increased by 1.3% annually in those aged younger than 50 years [2]. Chronic inflammation is a crucial pathogenic mechanism that accelerates CRC progression [3]. Inflammatory cells not only directly destroy intestinal epithelial cells but also facilitate genetic changes that drive carcinogenesis. Tumor inflammation signature also reflect adaptive immune response within tumors [4].

Epigenetically regulated gene expression profiles play an important role in colorectal cancer [5]. Circular RNAs (circRNAs) are conserved single-stranded RNA molecules derived from exonic or intronic sequences through premRNA back-splicing [6]. Unlike mRNA/lncRNA, they lack a 5′ cap and 3′ poly(A) tail, and therefore, are resistant to exonuclease digestion [7]. By competing with miRNAs, circRNAs can regulate gene expression and affect disease development. Previous studies have shown that circRNAs can function as biomarkers for various diseases and tumors [8–11]. Overexpression of circHEXTD1 can promote HuR-dependent autophagy via miR-182-5p and reduce colonic injuries and inflammation [12]. Analysis of differentially expressed genes between noninflammatory mucosa and inflamed mucosa showed that hsa_circ_0007919 regulates target genes by binding to hsa-let-7a and hsa-miR-138, affecting the clinical features and epithelial integrity of UC disease [13]. Inflammatory bowel disease (IBD), especially ulcerative colitis (UC), has been considered a typical pathogenic factor [14]. Twenty-one percent of IBD patients develop tumors within 10 years [15]. Colorectal cancer associated with colitis can progress from inflammation to dysplasia and eventually into tumors [16]. The expression of circRNA is significantly different in colorectal adenoma compared with adjacent normal tissues [17]. CircRNA_0000392 can act as a sponge for miR-193a-5p and regulates the expression of PIK3R3, affecting the proliferation and invasion of CRC cells [10]. There are a considerable number of studies on molecules/cells involved in the transition from inflammation to CRC. The AOM-DSS animal model for this disease is a method to objectively replicate human CAC and has been a great benefit for research [18]. However, the mechanism, such as the circRNA involved in the process of “inflammatory-CRA-CRC”, remains to be clarified, as there is limited information available.

The aim of this study was to reveal the circRNA expression profile in different tissues by constructing a mouse dynamic progression model of colorectal enteritis, colorectal adenoma and colorectal cancer to identify potential biomarkers in the process of malignant transformation of the disease. The circRNAs found in mouse models were transformed into human genomes, potential circRNAs were validated in human clinical tissue samples, and predictive biomarkers and potential therapeutic targets of CRC were identified. The diagnostic value of potential circRNAs was evaluated, and circRNA-associated ceRNA networks were constructed. Our results may provide new insights into the pathogenesis of CRC and provide possible candidate biomarkers for the early diagnosis of CRC.

## MATERIALS AND METHODS

### Animal experimental design

Six-week-old female wild-type C57BL/6 mice were purchased from Shanghai SLAC Laboratory Animal Co. Ltd, China. All mice were housed in an SPF environment and randomized into groups of five individuals per cage. The azoxymethane (AOM)-dextran sodium sulfate (DSS) model was described in previous studies [19]. All mice were treated with intraperitoneal injections of 12.5 mg/kg AOM (MP-USA). One week later, 2.5% DSS (MP-USA) was prepared in a bottle for mice to drink for 5 days. Then, the mice were allowed to drink distilled water for 16 days. The experiment ended when 4 cycles of the drinking protocol was completed. The body weight and water intake of the mice were tracked twice a week. Data regarding the shape of the intestine, spleen weight and polyp/tumor number and size were collected. Colon tissue was collected at −80°C and formalin-fixed for storage.

### Histology analyses and immunohistochemistry

Mouse colons were cut into 5 μm tissue sections for staining with hematoxylin and eosin when fixed and embedded. Glycan was detected with Periodic acid-Schiff (PAS) (Sigma–Aldrich), and general intestinal carbohydrate moieties were detected with Alcian blue (Sigma–Aldrich).

### RNA extraction, RNA sequencing data acquisition and processing, and qRT-PCR

TRIzol (Life Technologies CA, USA) was used to extract total RNA from colon tissues, including mouse and human tissues. We assessed the RNA quality via spectrophotometry and denaturing agarose gel electrophoresis. The circRNA sequencing data were acquired and analyzed as described previously [10]. STEM analysis [20] was used to identify the signature circRNA with persistent upregulation/downregulation. Samples of cDNA were synthesized from 1 mg of total RNA with reverse transcriptase (TaKaRa Biotechnology, Otsu, Japan). Total RNA was incubated with 5 U/μg RNase-R for 20 min at 37°C to degrade the linear RNAs. The expression of circRNA was detected using a SYBR Premix Ex Taq kit (TaKaRa Biotechnology). The qRT-PCR primers we used are listed in Table 1.

**Table 1:** List of each primer sequence.

**circRNA**	**Forward**	**Reverse**
hsa_circ_0101338	TTGGTTGCACATGGCAGGAT	ACGCCCAGGTTACTTACGTTT
hsa_circ_0033474	TTGGTTGCACATGGCAGGAT	CCCATCTCCATGTGACGTGT
hsa_circ_0033473	TACAGGCAGAGAGGTTGCAATA	CTTCTTGACGCCCATCTCCAT
hsa_circ_0033472	CCTTACAGGCAGAGAGGTTGC	CCCATCTCCATGTGACGTGT
hsa_circ_0033471	CCTCCTGTGCAGATGAACAACCTC	GCCTCACGGAGCCAAGATGG
hsa_circ_0008780	CTTACAGGCAGAGAGGTTGCAA	GCCCTTGTATCTACCAGGCAGAG
hsa_circ_0022429	TGGCCAATTAACTGAGAATGTAAGA	TCGGGAATCTCGGTCACAAC
hsa_circ_0022428	TGGCCAATTAACTGAGAATGTAAG	GAGCCACTACCCTGCCAGTT
hsa_circ_0022427	TGGCCAATTAACTGAGAATGTAAGA	CCGACTGCAGGTTGTCAAAG
hsa_circ_0022426	GCACTTTAATGGCCAATTAACTGA	GGAGTACCAGCGCATCTACACCA
mmu_circ_0000420	CCTCACATCGGAAACTACAGACTG	ATCCGAACTCCATTCAGTCTTGTC
CHR16:33798933-33799094+	GTCCTCCCACCACGGTACA	CTTTGTACTGTGGTGGGAGGAC
mmu_circ_0000698	CTGTGGATGACAGTGCAGAATTTA	ACATACTTGCCAGCATCGCTTT
mmu_circ_0008035	AGGGTCCTGAAGTTGATGTGGA	GGGTCCCAAGTTCAAGATGCC
CHR4:65155617-65156679+	AGAGTGCTGTGACCCAGACAT	AGCGTGGATCTCTGTTGCCTT
mmu_circ_0001769	GCTAGTGAGGAATGGAGCCAAG	ACTCCATTCAGTCTTGTCTCCCA
mmu_circ_0015847	TCCTGCCATTTCACTCTGTCA	TGCCAAACGAGCAGTCTCAC

### Functional enrichment and PPI analysis

GO and KEGG analyses were performed to detect the persistent upregulation/downregulation circRNA-associated genes. GO analysis was performed to describe three domains: biological processes, cellular components and molecular functions. (http://www.geneontology.org). The web-based Molecular Annotation System 3.0 (MAS 3.0) was used for pathway analysis, which integrated three different open-source pathway resources: KEGG, BioCarta and GenMAPP. The significantly altered pathways were selected using the threshold of the *p* value and FDR (corrected *p* value) < 0.05 derived from the hypergenomic test.

### Homology analysis of human and mouse circRNAs

The sequence of mouse circRNA was obtained and extracted from the results of our high-throughput sequencing, and then the sequence of human circRNA (HG19) was downloaded from circBase, and the homology analysis of mouse circRNA and human circRNA was performed using BLASTN 2.12.0+.

### Patient tissue samples

We collected samples from Longhua Hospital affiliated with Shanghai University of Traditional Chinese Medicine (Shanghai, China). The sample cohort included 16 CRA patients, and 16 CRC patients. This study was approved by the Ethics Committee of Longhua Hospital, and all patients signed informed consent forms. A total of 48 samples were collected (16 colorectal adenoma, 16 CRC tissues, and 16 adjacent normal tissues) from patients who underwent surgical treatment in Longhua Hospital, Shanghai, China. All resected specimens were snap frozen in liquid nitrogen and stored at −80°C. This study was approved by the Ethics Committee of Longhua Hospital, and informed consent was obtained from all participants.

### Analyses of circRNA-miRNA–mRNA interactions in CRC

Software programs, including circRNA Interactome and RegRNA, were used to predict circRNA-miRNA interactions. A set of miRNA target genes were predicted based on data from the miRanda, miRDB, miRWalk, RNA22 and TargetScan databases. Cytoscape software was used to construct the circRNA-miRNA–mRNA network.

### Fluorescent *in situ* hybridization analysis

Fluorescent *in situ* hybridization analysis [21] was performed using a fluorescent hybridization kit (C007, Gefan tech, China) to detect circRNA expression in different mouse intestinal tissues and human intestinal tissues. The FISH probes for mmu_circ_0000420, mmu_circ_0008035, hsa_circ0101338 and hsa_circ0022426 was designed and synthesized by (Jiman tech, China). Cell nuclei were stained with DAPI.

### Cell culture and transfection

HCT116 cells was obtained from the Stem Cell Bank, Chinese Academy of Sciences (Shanghai, China) and was cultured in McCoy’s 5A (Gibco, Carlsbad, CA, USA) with 10% (v/v) FBS. Lipofectamine 2000 (Invitrogen, CN2483146) transfection of the constructed siRNA (Shanghai Jiiman Biotechnology Co., hsa-circ-0101388:5′-3′, BIOTIN-ACCAATGTAAACGCTGTAGTTCT; has-circ-0022426:5′-3′, BIOTIN-GTACATCCCCGCTTCAAGGTTAA) was transfected into HCT116 cells. Negative control siRNAs showed no significant sequence similarity to mouse, rat, or human gene sequences. Controls were previously tested in a cell-based screen and demonstrated no significant effect on cell proliferation, viability or morphology. After 4–6 hours the cell culture medium was replaced with normal medium and after 48 hours micRNA was extracted with the kit (QIAZOL, QIAGEN Sciences, LOT 1023537; miRNeasy Mini Kit, QIAGEN Sciences, LOT 172011276) and reverse transcribed for qRT-PCR (Table 2) validation.

**Table 2:** List of primer sequences.

**miRNA**	**Sequence**
hsa-miR-1273h-5p	CGGGCCTGGGAGGTCAAGG
hsa-miR-6774-5p	CGGGCACTTGGGCAGGAGGGACC
hsa-miR-619-5p	CGGGCGCTGGGATTACAGGC
hsa-miR-659-5p	CGGGCAGGACCTTCCCTGAA
hsa-miR-1273g-3p	CGGGCACCACTGCACTCCA
hsa-miR-6774-5p	CGGGCACTTGGGCAGGAGGGACC
hsa-miR-635	CGGGCACTTGGGCACTGAAAC
hsa-miR-6851-3p	CGGGCTGGCCCTTTGTACC
hsa-miR-1227-5p	CGGGCGTGGGGCCAG
hsa-miR-661	CGGGCTGCCTGGGTCTCTGGCC
hsa-miR-1273h-5p (JH)	CCTGTTGTCTCCAGCCACAAAAGAGCACAATATTTCAGGAGACAACAGGACTGCAG
hsa-miR-6774-5p (JH)	CCTGTTGTCTCCAGCCACAAAAGAGCACAATATTTCAGGAGACAACAGGCATACAG
hsa-miR-619-5p (JH)	CCTGTTGTCTCCAGCCACAAAAGAGCACAATATTTCAGGAGACAACAGGGGCTCAT
hsa-miR-659-5p (JH)	CCTGTTGTCTCCAGCCACAAAAGAGCACAATATTTCAGGAGACAACAGGTCCTTGG
hsa-miR-1273g-3p (JH)	CCTGTTGTCTCCAGCCACAAAAGAGCACAATATTTCAGGAGACAACAGGCTCAGGC
hsa-miR-635 (JH)	CCTGTTGTCTCCAGCCACAAAAGAGCACAATATTTCAGGAGACAACAGGGGACATT
hsa-miR-6851-3p (JH)	CCTGTTGTCTCCAGCCACAAAAGAGCACAATATTTCAGGAGACAACAGGCTGGAGG
hsa-miR-4755-3p (JH)	CCTGTTGTCTCCAGCCACAAAAGAGCACAATATTTCAGGAGACAACAGGACTTTCC
hsa-miR-1227-5p (JH)	CCTGTTGTCTCCAGCCACAAAAGAGCACAATATTTCAGGAGACAACAGGCCACCGC
hsa-miR-661 (JH)	CCTGTTGTCTCCAGCCACAAAAGAGCACAATATTTCAGGAGACAACAGGACGCGCA
universal—R	Reverse: CAGCCACAAAAGAGCACAAT

### Statistics

Statistical analysis was performed using GraphPad Prism 7.0 (GraphPad Software, Inc., CA, USA). Student’s *t* test and one-way ANOVA were used to compare differences between groups. Statistical significance was defined as *p* < 0.05. The area under the ROC curve was used to assess the predictive value of circRNA in the diagnosis of CRA/CRC.

## RESULTS

### The AOM-DSS model recapitulated progression and showed an increased number of polyps/tumors

**Figure 1:**
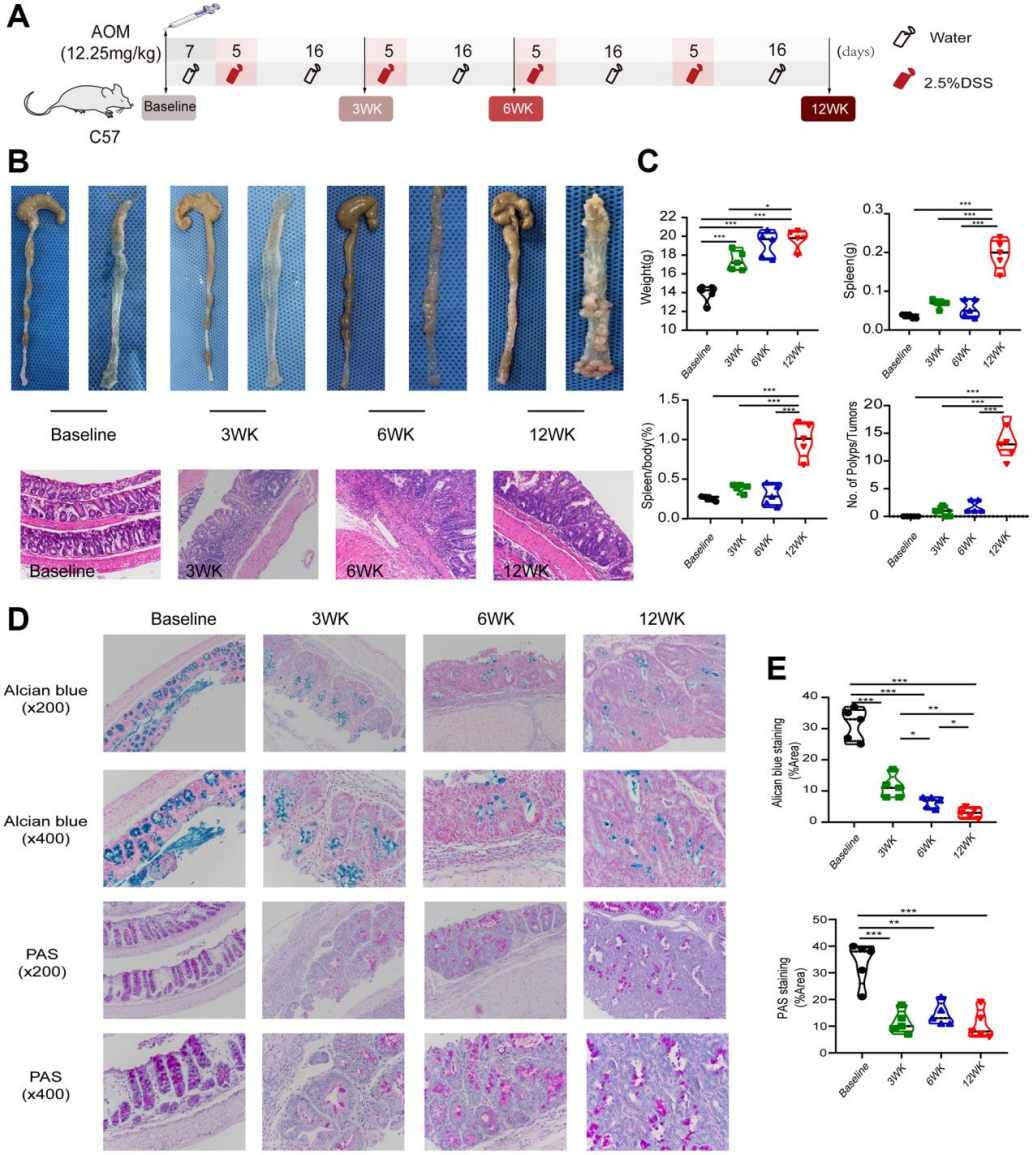
**The AOM-DSS model recapitulated “inflammation -CRA-CRC” progression.** (**A**) Schematic representation of the study design. The intestinal conditions and disease progression of mice were evaluated at baseline and 3 weeks, 6 weeks and 12 weeks after intervention. (**B**) Representative images and H&E staining images (original × magnification 200) of the colon. (**C**) Body weight, spleen weight, spleen/body (%) change, and number of polyps/tumors in different groups. (**D**) Alcian blue staining and PAS staining. (**E**) Alcian blue and PAS staining areas and fluorescence intensity quantified by ImageJ based on different groups. ^*^p < 0.05; ^**^p < 0.01; ^***^p < 0.001.

We first induced a mouse model of inflammation-based tumorigenesis using AOM and DSS (Figure 1A). The intestinal conditions of the mice were observed at the start of the study and at 3 weeks, 6 weeks and 12 weeks after treatment. During the study, the size and number of gross polyps increased substantially (Figure 1B). In addition, compared with healthy baseline mice, AOM-DSS-treated mice exhibited a progressive increase in spleen-body ratio (Figure 1C). Histological analysis showed that the AOM-DSS model reproduced the progression from chronic inflammation to colorectal adenoma to colorectal carcinoma *in situ* at different time points (Figure 1D). Further pathological staining analysis showed that steady-state molecules were gradually reduced and that mucosal integrity was gradually lost during the inflammatory tumorigenesis process (Figure 1E). These results indicated that the model of inflammation-based tumorigenesis in mice was successfully constructed and provided a basis for the transcriptome study of different pathological tissues in the progression of colorectal cancer.

### Identification of circular RNAs expressed at different stages in inflammation-based tumorigenesis using RNA-Seq analyses

**Figure 2:**
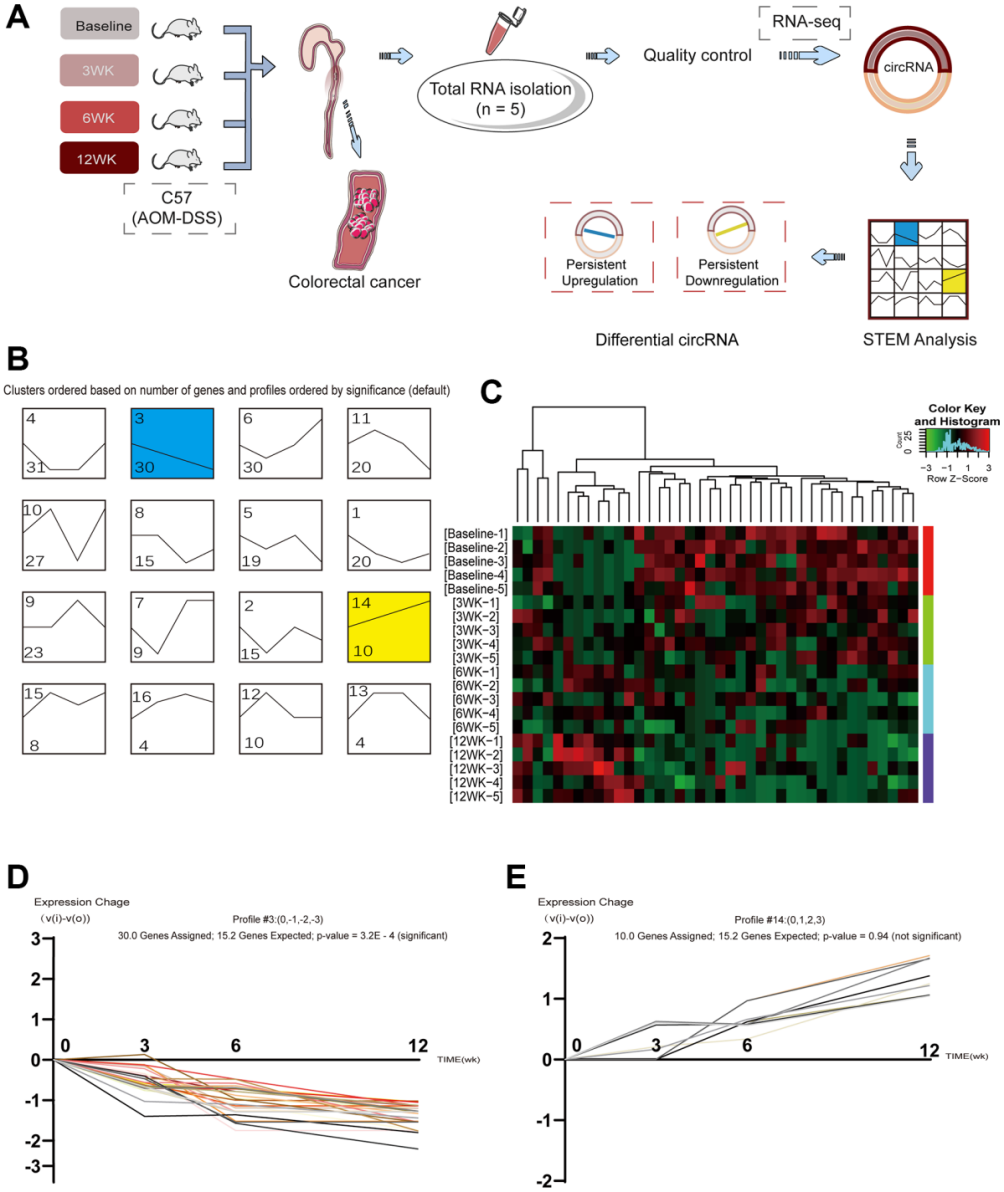
**Identification of circular RNAs expressed at different stages in inflammation-based tumorigenesis.** (**A**) Schematic representation of circRNA identification and sequencing; (**B**) Cluster based on changes in circRNA; (**C**) Heatmap of the clustering analysis indicating differences in circRNA expression profiles in the stage of “inflammation-CRA-CRC”. Areas colored red and green indicate the upregulation and downregulation of circRNA, respectively; (**D**) circRNAs with persistent downregulation; (**E**) circRNAs with persistent upregulation.

To identify circRNAs differentially expressed during inflammation-based tumorigenesis, we analyzed circRNA expression using secondary sequencing (Figure 2A) in colorectal tissue samples from each group of five mice at four time points: at baseline and 3, 6, and 12 weeks after treatment. A total of 14,973 circRNAs were detected in the colorectal tissues, and the list of total circRNA expression profiles is shown in Additional Files 1 and 2. STEM analysis, a tool to cluster and compare short time series gene expression data, was performed on four sets of data to identify the continuous changes in circRNA expression during the dynamic course of disease progression. We found that there were significantly differentially expressed circRNAs under 16 different dynamic trends (Figure 2B). Cluster analysis showed that with the progression of the disease, 30 circRNAs were downregulated, while 10 circRNAs were upregulated (Figure 2C–2E). The results of the analysis provide evidence that there are continuously varying and differentially expressed circRNAs in inflammation-based tumorigenesis, and these continuously varying circRNAs have the potential to become biomarkers for disease prediction.

### GO and KEGG analyses of circRNAs with persistent downregulation/upregulation

**Figure 3:**
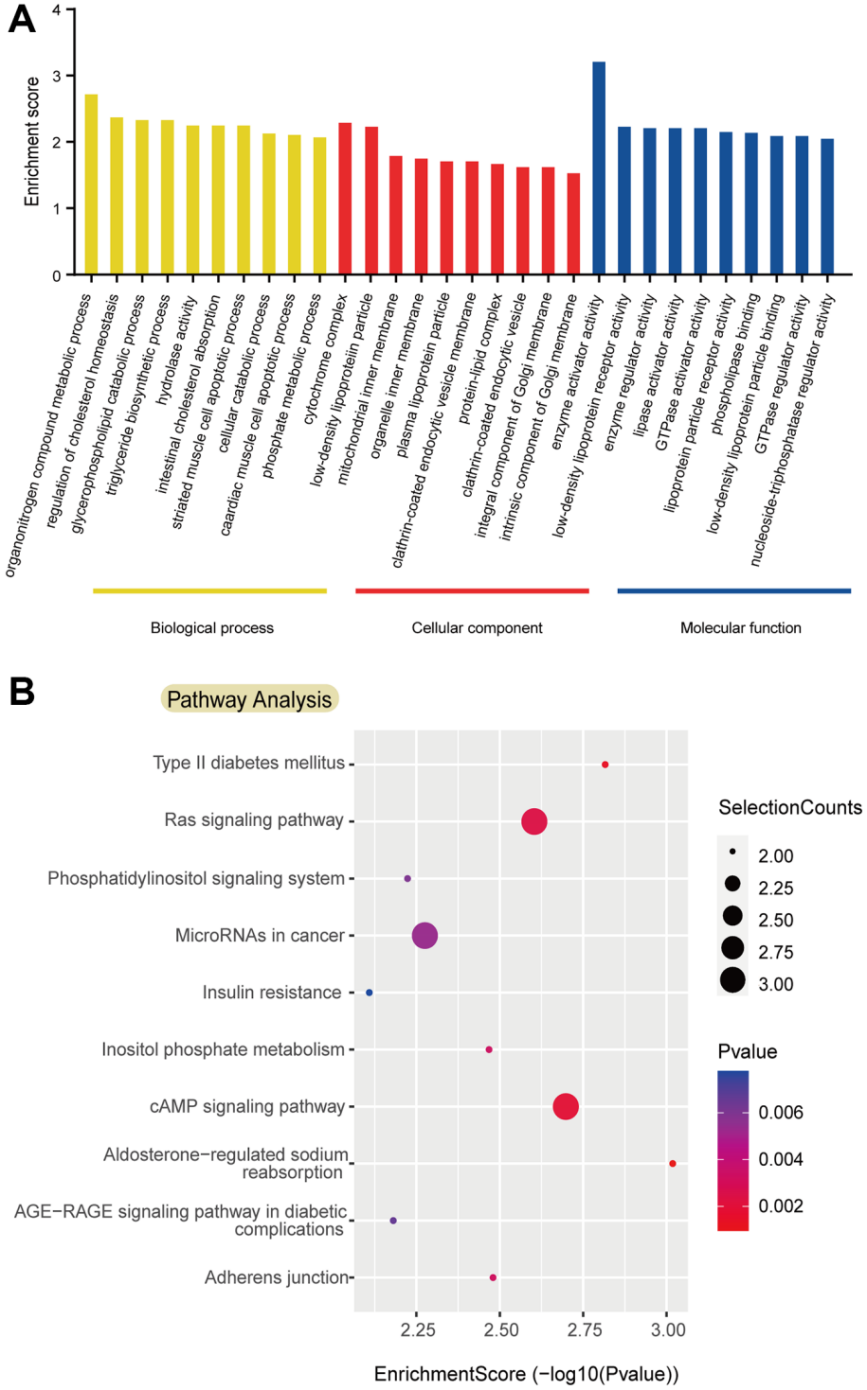
**Gene ontology and kyoto encyclopedia of genes and genomes pathway analyses based on persistent downregulation/upregulation.** (**A**) Top 10 Gene Ontology (GO) terms identified in GO analysis; (**B**) Top 10 pathways identified in Kyoto Encyclopedia of Genes and Genomes pathway analysis. Abbreviations: GO: Gene Ontology; KEGG: Kyoto Encyclopedia of Genes and Genomes.

Functional annotation GO enrichment analysis of persistent upregulation/downregulation of circRNAs comprises three parts: cellular component (CC), biological process (BP), and molecular function (MF). The top ten terms were enumerated in the related functional area (Figure 3A). KEGG pathway enrichment analysis revealed the 10 pathways among persistently altered circRNAs. It is noteworthy that the “Ras signaling pathway”, “microRNAs in cancer” and “cAMP signaling pathway” play important roles in the disease process (Figure 3B).

### Verification of differentially expressed circRNA by qRT–PCR and FISH experiment

**Figure 4:**
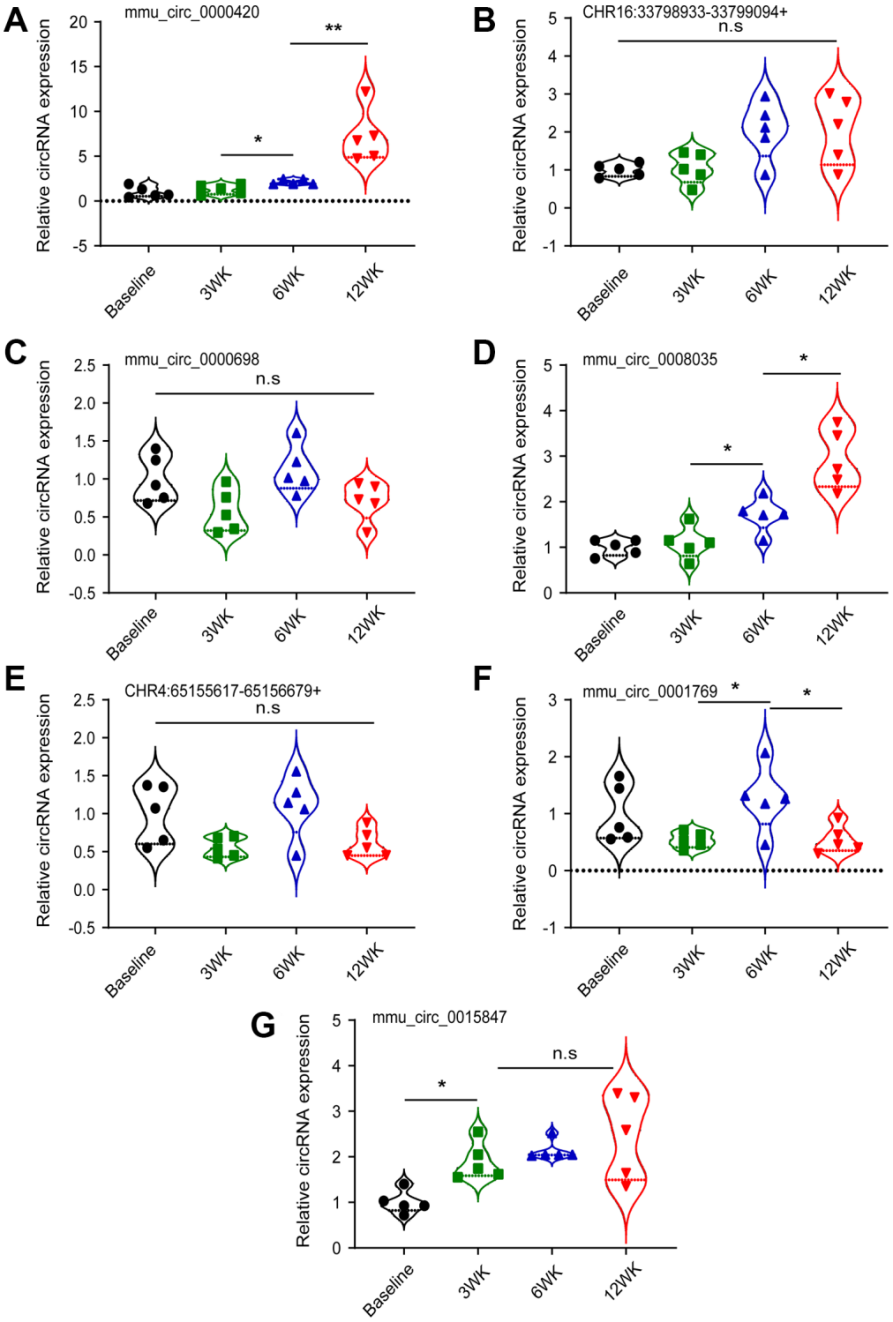
**Validation of candidate circRNAs by qRT-PCR.** (**A**) mmu_circ_0000420; (**B**) CHR16:33798933-33799094+; (**C**) mmu_circ_0000698; (**D**) mmu_circ_0008035; (**E**) CHR4:65155617-65156679+; (**F**) mmu_circ_0001769; (**G**) mmu_circ_0015847. ^*^p < 0.05; ^**^p < 0.01; ^***^p < 0.001.

Based on the results of circRNA sequencing and STEM analysis, we focused on circRNAs that continued to rise based on the principle of finding biomarkers for disease progression, and combined with the basic expression level of circRNAs, finally screened out 7 circRNAs whose expression continued to rise in disease progression for subsequent validation analysis: mmu_circ_0000698, mmu_circ_0001769, mmu_circ_0008035, mmu_circ_0000420, mmu_circ_0015847, CHR4:65155617-65156679+, CHR16:33798933-33799094+. qRT-PCR showed that mmu_circ_0008035 and mmu_circ_0000420 (Figure 4) were statistically significant in both stages of “inflammation-CRA” (*p* < 0.05, *p* < 0.01) and “CRA-CRC” (*p* < 0.05, *p* < 0.05). FISH experiments further confirmed that the expression of these two potential targets increased in mouse colorectal tissue as the disease progressed (Supplementary Figure 1).

### Homology analysis of human and mouse circRNAs

**Figure 5:**
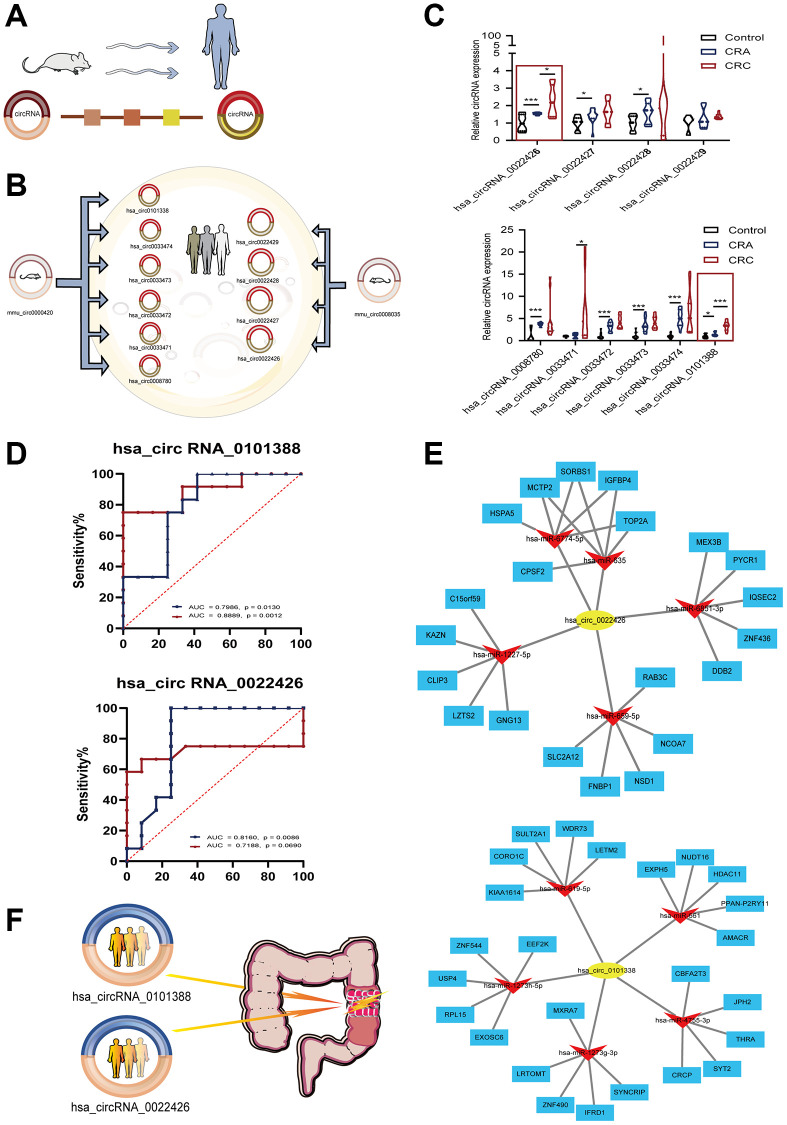
**Candidate circRNAs in human.** (**A**) Homology analysis schematic of human and mouse circRNAs; (**B**) six circRNAs corresponding to mmu_circ_0000420 and four circRNAs corresponding to mmu_circ_0008035 in human; (**C**) qRT-PCR of circRNAs in human; (**D**) ROC of hsa_circ0101338 and hsa_circ0022426; Blue dotted lines represent diagnostic values for discriminating CRA from normal colon tissue; Red dotted lines represent diagnostic values for discriminating CRC from CRA; (**E**) circRNA-miRNA-mRNA network analysis of hsa_circ0101338 and hsa_circ0022426; (**F**) hsa_circ0101338 and hsa_circ0022426 can be early predictive biomarker of CRC. ^*^p < 0.05; ^**^p < 0.01; ^***^p < 0.001.

For the two circRNAs with significant changes in disease progression in the animal models, we conducted an analysis of circRNAs homology in humans and mice in the database. The circRNAs in the human genome corresponding to two circRNAs in mice were analyzed, including six circRNAs corresponding to mmu_circ_0000420: hsa_circ0101338, hsa_circ0033474, hsa_circ0033473, hsa_circ0033472, hsa_circ0033471, and hsa_circ008780 and four circRNAs corresponding to mmu_circ_0008035 in humans: hsa_circ0022429, hsa_circ0022428, hsa_circ0022427, and hsa_circ0022426. (Figure 5A, 5B).

### Verification of differentially expressed circRNAs in humans by qRT-PCR and FISH experiment

Next, we used colorectal adenoma and tumor samples to analyze circRNA expression. HE staining confirmed the intestinal pathological status of CRA samples, CRC samples and paracancer samples (Supplementary Figure 2). Among the circRNAs expressed in human colon tissue, only hsa_circ0101338 and hsa_circ0022426 (Figure 5C) exhibited a statistically significant change in CRA compared to normal tissue (*p* < 0.05, *p* < 0.01). Again, statistical significance was confirmed in CRC compared to CRA (*p* < 0.01, *p* < 0.05). These two circRNAs play important roles in the “inflammation-CRA-CRC” process. Fish experiment also confirmed the expression of hsa_circ0101338 and hsa_circ0022426 in colorectal adenoma and colorectal tumor tissues. The expression of hsa_circ0101338 was significantly stronger in CRC tissues than in CRA (Supplementary Figure 3). ROC analysis was used to evaluate their diagnostic value (Figure 5D). The area under the curve (AUC) of hsa_circ0101338 was 0.7986 and 0.9792 in CRA and CRC, respectively. The AUC of hsa_circ0022426 was 0.816 and 0.9063 in CRA and CRC, respectively. These results suggested a good positive diagnostic value of hsa_circ0101338 and hsa_circ0022426 in CRA and CRC (Figure 5F). Therefore, the above two circRNAs can be used as early predictive biomarkers of CRC.

### ceRNA network

A ceRNA is one of the mechanisms of circRNA; that is, circRNA acts as a “miRNA sponge” to regulate target genes. We made several unexpected observations in the results of the analysis. Thus, Cytoscape software was used for further analysis of the circRNA-miRNA–mRNA regulatory networks to clarify the underlying mechanism of hsa_circ0101338 and hsa_circ0022426. As described in Figure 5E, the top 5 miRNAs that may bind to potential circRNAs and the 5 most likely target genes of each miRNA are displayed, hsa_circ_0022426: hsa-miR-6774-5p, hsa-miR-635, hsa-miR-6851-3p, hsa-miR-1227-5p, hsa-miR-659-5p; hsa_circ_0101338: hsa-miR-619-5p, hsa-miR-661, hsa-miR-1273h-5p, hsa-miR-1273g-3p, hsa-miR-4755-3p. From the prediction results, we selected the top 5 candidate miRNAs to validate the specific interaction by RNA pull-down assay. The results showed that the expression level of miR-635, miR-6851-3p, miR-1227-5p, miR-6774-5p was significantly reduced in the circ_0022426 probe compared with the oligo probe in HCT -116 cells (*p* < 0.01); the expression of miR-661, miR-4755-3p, miR-1273g-3p was reduced in the circ_0101338 probe compared with the oligo probe. (*p* < 0.01) (Supplementary Figure 4). Overall, these results demonstrate that circ_0022426 acts as a sponge for miR-635, miR-6851-3p, miR-1227-5p, miR-6774-5p in CRC cells, while circ_0101338 acts as a sponge for miR-661, miR-4755-3p, and miR-1273g-3p in CRC cells.

## DISCUSSION

Inflammation is one of the key pathological bases mediating the development of tumors, and inflammatory cancer transformation is the key pathological stage of normal cell malignant transformation [22]. Colorectal cancer is a classic inflammatory-dependent tumor, and most colorectal cancers develop from advanced inflammatory adenomas [23, 24]. Therefore, for colorectal cancer, identifying early predictive biomarkers of adenoma-to-malignant tumor transformation and exploring the potential molecular characteristics of the disease are important areas of research. CircRNAs [25] are members of the family of noncoding RNAs that form a closed continuous loop by covalently attaching to the 3′ and 5′ ends. Because of these unique structural properties, circRNAs have become promising diagnostic markers and therapeutic targets for cancer. To date, many studies have reported that circRNAs play an important regulatory role in the occurrence and development of human diseases, especially in the malignant progression of a variety of cancers [10, 26]. Therefore, we explored which circRNAs might be involved in the transition from benign to malignant features in colorectal cancer.

In this study, an induced model of colorectal cancer in mice was established through intraperitoneal injection of AOM combined with administration of drinking water containing DSS [27, 28]. Dynamic observations were made during the course of the disease. Pathological assessment of colon tissue was performed at 3, 6, and 12 weeks after DSS intervention. As time progressed, the intestinal tract of mice gradually changed from inflammation to benign adenoma until the intestinal tract of mice developed malignant tumor lesions at the 12^th^ week after DSS intervention, confirming the successful establishment of the model of intestinal inflammatory cancer transformation.

To investigate which circRNAs may be involved in the inflammatory transformation of colorectal cancer, high-throughput sequencing was performed on intestinal tissue samples from mice at baseline and 3, 6, and 12 weeks after intervention to investigate the association between circRNAs and malignant transformation in CRC. A total of 14973 circRNAs were detected in the colorectal tissues of all the groups. Then, STEM analysis was used to identify circRNAs with expression levels that continued to change during disease development in the four groups of tissue samples. Based on the results of the STEM analysis, we found 10 circRNAs with significantly increased continuous expression during the transformation of CRC. Through subsequent pathway enrichment analysis, we found that the circRNAs with continuously increased expression during tumor progression were mainly involved in the RAS signaling pathway and cAMP signaling pathway. In addition, we hypothesized that these 10 differentially expressed circRNAs may play important regulatory roles in the malignant transformation of colorectal cancer. We focused on 7 of the 10 circRNAs and further verified the accuracy of our sequencing results by qRT-PCR detection. Among them, further analysis was performed to confirm that the expression levels of mmu_circ_0000420 and mmu_circ_0008035 continued to increase significantly. Fish analysis confirmed the increased expression of the two significant circRNAs accompanies the disease process. Based on the results, we believe that mmu_circ_0000420 and mmu_circ_0008035 play key regulatory roles in the development of colorectal tumors in a mouse model.

The expression of circRNAs in a mouse model was verified, and potential circRNAs with important contributions to disease development were found. Next, the ultimate goal of this study was to identify important circRNAs involved in the malignant transformation of human CRC and provide biomarkers for the early prediction of the malignant transformation of human CRC. The genomic data in mice exhibit a high degree of similarity with the human genome data. CircRNAs are abundant in mammalian tissues and have certain conserved sequences, and most circRNAs are expressed simultaneously in humans and mice [29]. For the two circRNAs with significant changes in disease progression in the animal models, we conducted an analysis of circRNAs homology in humans and mice in the database. The circRNAs in the human genome corresponding to two circRNAs in mice were analyzed, including six circRNAs corresponding to mmu_circ_0000420 and four circRNAs corresponding to mmu_circ_0008035 in humans. Based on the results of this data comparison, we identified candidate human circRNAs with different changes in the malignant transformation process of colorectal cancer. Then, we verified whether these potential circRNAs were expressed differently in the malignant transformation of human CRC and whether they can be used as biomarkers for the early prediction of the occurrence of CRC in clinical tissue samples. In the validation of clinical samples, it was found that the expression levels of hsa_circRNA_0101388 and hsa_circRNA_0022426 continued to increase significantly in the clinical cohort at different stages of the malignant transformation of colorectal cancer, and ROC analysis also showed that these two circRNAs had potential diagnostic value for the malignant transformation of CRC. There is a lack of biomarkers for the early diagnosis and prediction of the inflammatory transformation of colorectal cancer. In recent years, through continuous exploration, researchers have found some potential biomarkers for the prediction of colorectal cancer from the perspective of intestinal microbiota [30, 31]. At the transcriptome level [32], the important roles of some potential oncogenes and tumor suppressor genes in the transformation of CRC from inflammatory adenoma to colorectal cancer have also been gradually revealed. As members of the noncoding RNA family, circRNAs are also involved in the development of a variety of tumors. In the occurrence and development of colorectal cancer, the functions and mechanisms of various circRNAs have been reported [33]. Studies have shown that hsa_circRNA_102610 plays an important role in the transformation from inflammatory bowel disease to CRC through the regulation of the miR-130a-3p-TGF-β1 axis [34]. In our previous study, we revealed the expression profile of circRNAs associated with liver metastasis of colorectal cancer and identified circRNAs with early prediction potential for liver metastasis of colorectal cancer [26]. Furthermore, the specific mechanism of one circRNA in the progression and metastasis of colorectal cancer was revealed [10]. In this study, we found that two potential circRNAs have potential early predictive value in the development of colorectal adenoma and malignant transformation to CRC.

A number of previous studies on the function of circRNAs have shown that circRNAs act as miRNA sponges and regulate the expression of downstream target genes, which is one of the important ways that circRNAs impact biological functions [35–37]. Therefore, for the two circRNAs with potential diagnostic value identified in our study, we predicted their possible sponge miRNAs and their downstream regulated target mRNAs by analyzing different databases. The potential regulatory mechanism of circRNAs was explored by constructing a ceRNA regulatory network. The ceRNA network established in this study provides a scientific basis for subsequent research on the mechanism of circRNA in the malignant transformation of CRC. However, further studies are needed to explore the important factors affecting the development of tumors, such as the relationship between stroma [38], immune cells [39] and circRNA, and to further explore the occurrence and development of colorectal tumors. Although tumorigenesis is the result of genetic and epigenetic alterations accompanied by mutations in tumor cells, pathological changes in the stroma also contribute to tumorigenesis [40].

## CONCLUSION

In conclusion, our study was based on the analysis of circRNA expression levels in colorectal tissues of mice at different pathological stages of inflammatory cancer transformation, combined with the homology transformation of circRNA in mice and humans, and finally verified by clinical samples. We found that hsa_circRNA_0101388 and hsa_circRNA_0022426 have potential predictive value for the early emergence of colorectal cancer. Our results may provide new biomarkers for predicting the occurrence of colorectal cancer inflammatory cancer transformation. Validation of larger samples is necessary to confirm these findings, and subsequent studies will further explore the biological functions of these potential circRNAs in the development of CRC.

## Supplementary Materials

Additional File 1

Additional File 2

Supplementary Figures
