# Supplementary material for: The circRNA expression profile of colorectal inflammatory cancer transformation revealed potential predictive biomarkers
Source: Aging (Albany NY). 2022 Nov 29;14(22):9280–99. doi: 10.18632/aging.204406 (PMC9740358; doi:10.18632/aging.204406)
Supplement: Supplementary Figures [file aging-14-204406-s003.pdf]

SUPPLEMENTARY FIGURES

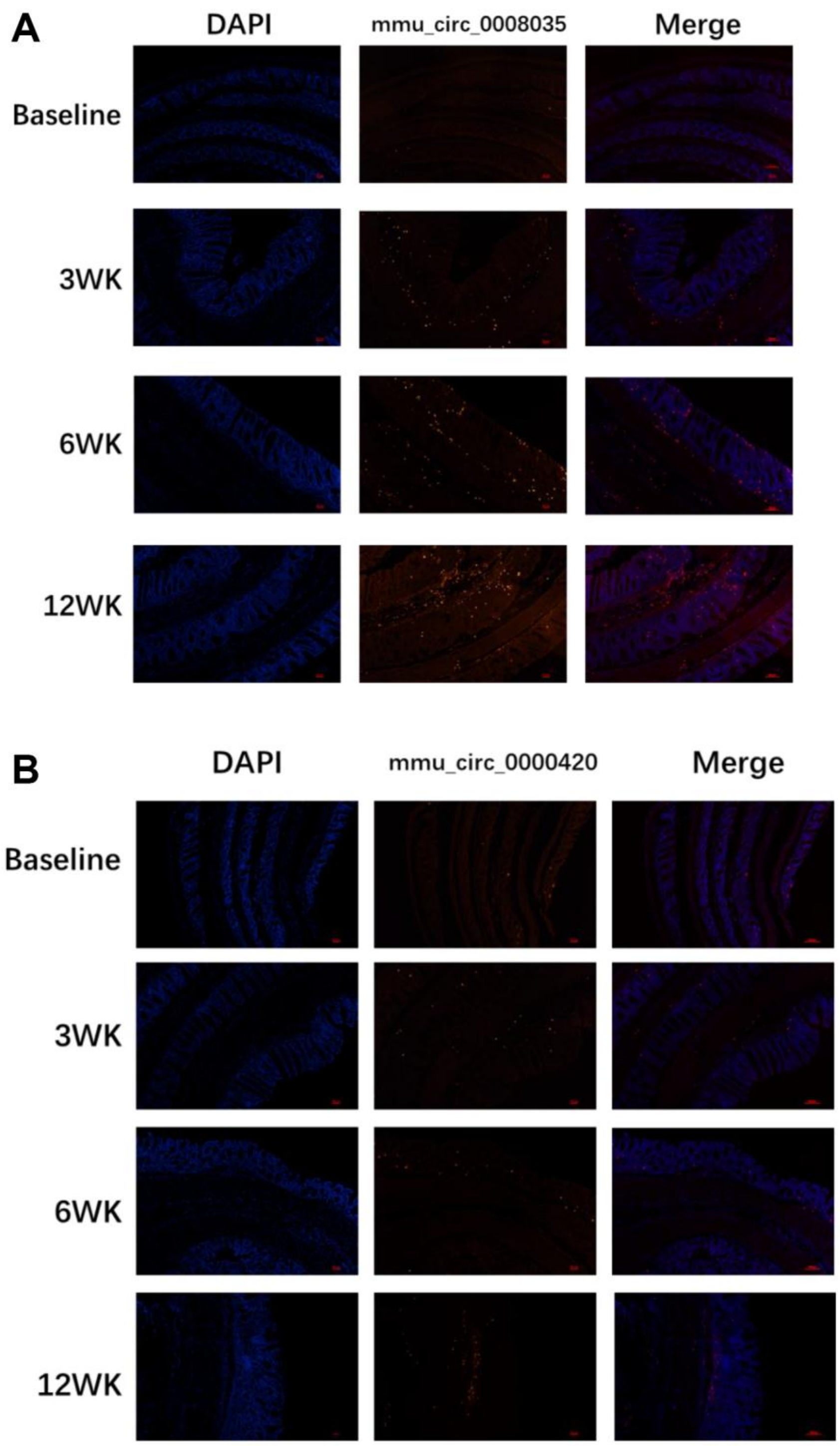

Supplementary Figure 1. Fish assay of circRNA in inflammation-based tumorigenesis. (**A**) The expression of mmu_circ_0008035 in mice colorectal tissues (X100); (**B**) The expression of mmu_circ_0000420 in mice colorectal tissues(X100).

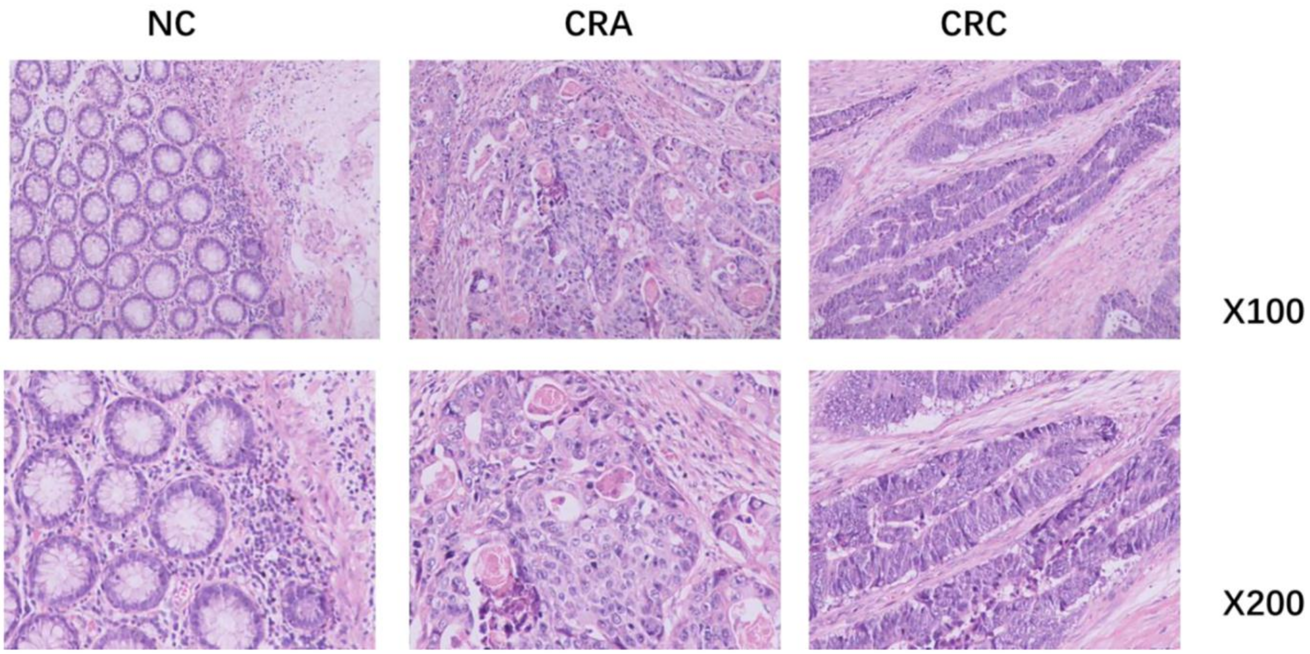

Supplementary Figure 2. HE staining of colorectal tissues from patients with paraneoplastic, colorectal adenoma, and colorectal tumors.

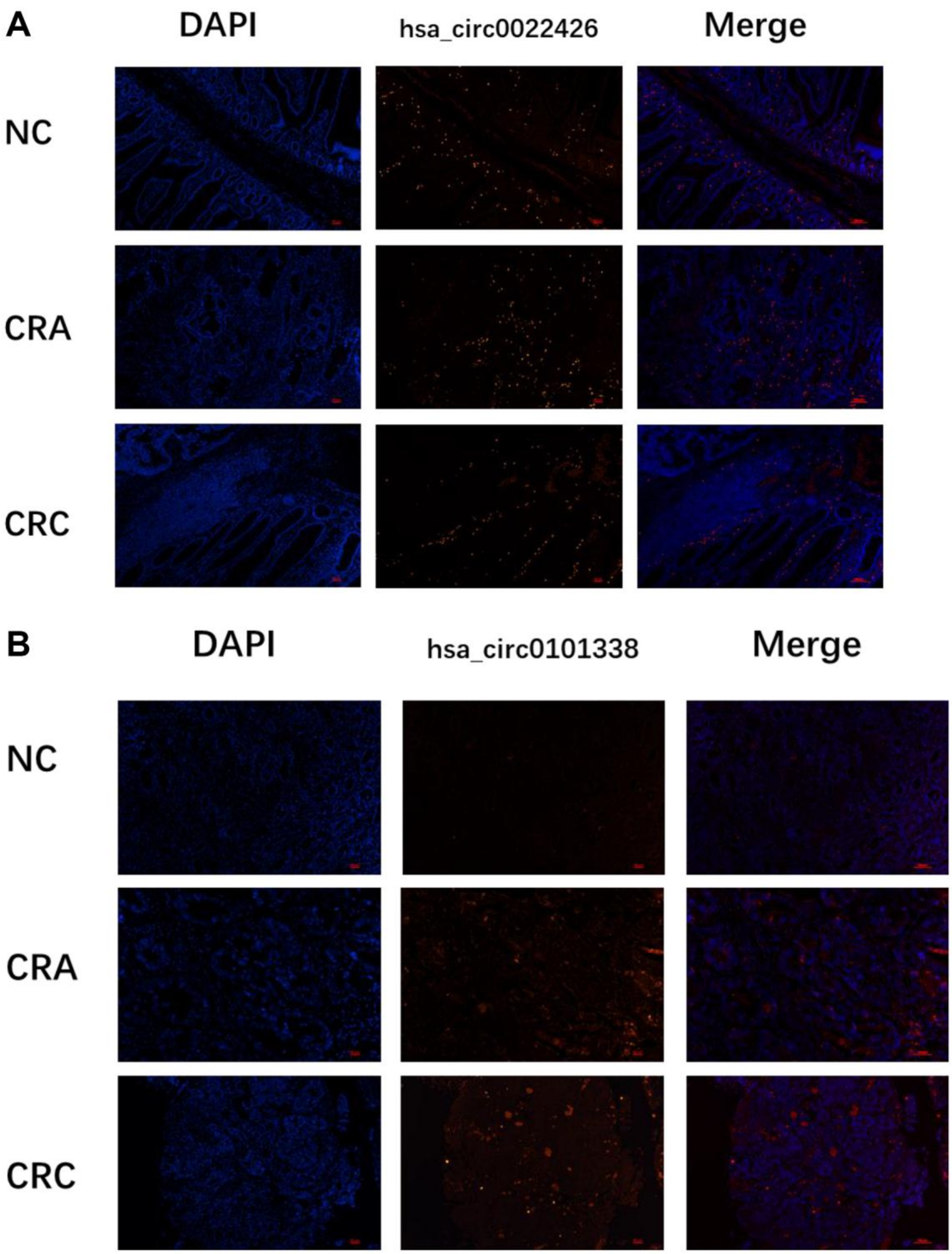

Supplementary Figure 3. Fish assay of circRNA in CRA and CRC patients. (**A**) The expression of hsa_circ0022426 in CRA and CRC patients(X100); (**B**) The expression of hsa_circ0101338 in CRA and CRC patients(X100).

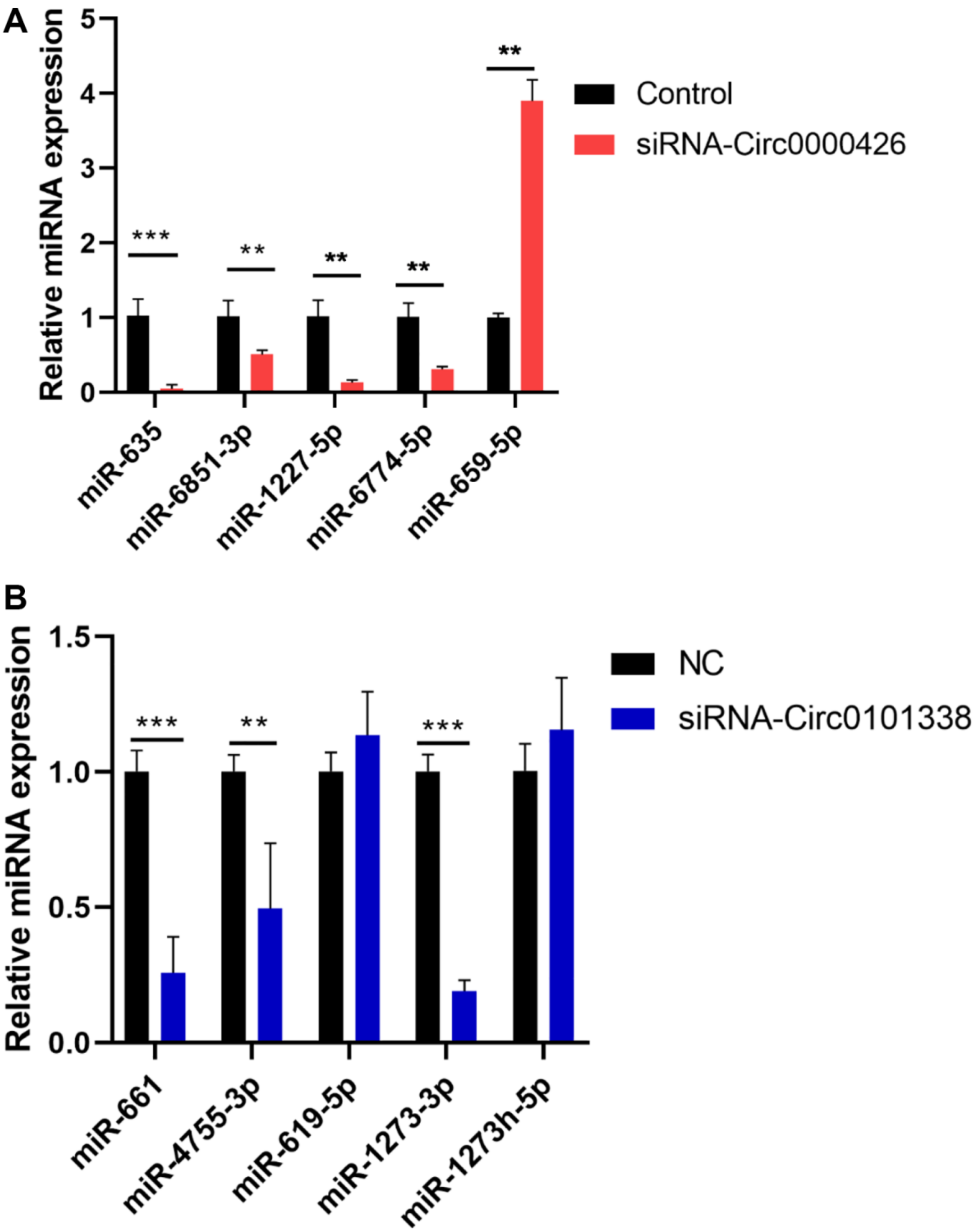

Supplementary Figure 4. The level of miRNA in HCT 116 after transfection with siRNA. (**A**) The expression of miRNA after transfection with hsa_circ0022426; (**B**) The expression of miRNA after transfection with hsa_circ0101338.
